# When peer review drags on: the harm to early career researchers

**DOI:** 10.3389/frma.2026.1740381

**Published:** 2026-02-04

**Authors:** Noemi Felisi, Riccardo Vecchio, Anna Odone

**Affiliations:** 1 Faculty of Public Health and Policy, London School of Hygiene and Tropical Medicine, London, United Kingdom; 2 Department of Infectious Diseases, Luigi Sacco Hospital, ASST Fatebenefratelli Sacco, Milan, Italy; 3 PhD National Programme in One Health Approaches to Infectious Diseases and Life Science Research, Department of Public Health, Experimental and Forensic Medicine, University of Pavia, Pavia, Italy; 4 Istituti Clinici Scientifici Maugeri IRCCS, Pavia, Italy; 5 Department of Public Health, Experimental and Forensic Medicine, University of Pavia, Pavia, Italy; 6 Medical Direction, Fondazione IRCCS Policlinico San Matteo, Pavia, Italy

**Keywords:** academic publication, early career researcher (ECR), peer review, publication delays, research equity

## Introduction

I am an infectious diseases resident and early career researcher (ECR) from Italy interested in global health. In August 2024, I submitted my first manuscript to a reputable peer-reviewed journal from a major publisher: an original article on barriers and facilitators to Cervical Cancer Screening with data I collected in Northern Uganda in the context of research fellowship for my medical school thesis. After months of fieldwork with peers and senior supervisors, I was excited to share our findings. I checked the submission web platform weekly and sent periodic reminders to the editorial office. Yet, the paper remained unassigned to an editor for more than a year (380 days)!

This experience is not merely anecdotal. Large-scale analyses have documented a global growing strain on the peer review system, characterized by prolonged review timelines, reviewer shortages, and increasing mismatches between submission volumes and reviewing capacity (Global State of Peer Review, 2018; Adam, 2025; Odone et al., 2020). In this Opinion piece, we argue that peer review delays disproportionately harm ECRs, affecting not only scientific progress but also confidence, career opportunities, and trust in the academic system. After outlining the structural drivers of the peer review crisis, we examine reform proposals and their potential to make peer review more sustainable, transparent, and equitable, especially for ECRs.

## The crisis of the peer review system

Peer review remains a cornerstone of scientific integrity. It ensures methodological rigor, improves reporting, and guides editorial decisions. However, the system is increasingly strained. A recent cross-sectional study of 57 health policy journals reported median submission-to- publication times ranging from 35 to 353 days, with time to first editorial decision reaching up to 67 days, and the peer review phase extending to 314 days (Phillips and Horn, 2025). Similar delays have been documented in primary care journals, where submission-to-publication lags average 243 days, with peaks of over one year (Chen et al., 2024). Delays in peer review reflect interacting constraints across multiple stakeholders, including authors, reviewers, editors, publishers, and academic institutions (Kadaifci et al., 2025; Kusumoto et al., 2023).

The efficiency of a journal's editorial office and administrative support plays a major role: high-impact and highly selective journals often exhibit faster turnaround times, while journals with limited editorial staff can introduce longer bottlenecks in review copyediting, proofing, and scheduling for publication. However, editorial constraints alone do not fully explain the scale of current delays.

A central challenge lies in securing peer reviewers. Editors must often send multiple invitations for each manuscript, as many are declined, ignored, or accepted but never completed (Peterson et al., 2022). In a 2016 Publons editor survey, 75% of editors identified “finding reviewers and getting them to accept review invitations” as the most difficult aspect of their role (Global State of Peer Review, 2018). At the heart of this crisis is a widening mismatch between demand and supply (Adam, 2025). Article submission and publication output have risen steadily, especially after the Covid-19 pandemic: for example, the number of peer reviewed public health articles increased by 25% between 2019 and 2020 and by a further 21% between 2020 and 2021 (Künzli et al., 2022). However, the pool of active reviewers appears stagnant or is even shrinking (Adam, 2025).

The most commonly cited reason for declining a review invitation is that the manuscript falls outside the reviewer's area of expertise. Increasing specialization across research fields narrows relevant expertise and complicates the identification of appropriate reviewers (Kusumoto et al., 2023). Time constraints represent another major barrier, as reviewing requires time and competes with researcher's own funded work (Beecher and Wang, 2025). The ongoing decline in secure academic positions has further eroded both the time and willingness of researchers to engage in unpaid service (Ellaway, 2024). As a result, the reviewing burden is concentrated among a small minority: within biomedical sciences, an estimated 20% of researchers perform up to 90% of reviews, intensifying fatigue and delay (Ellaway, 2024).

Additional delays arise once reviewers are secured. Reviewers may require extensions, and multiple rounds of revision can further prolong the process. Review times tend to decrease with increasing journal impact factor, despite reviews often being longer. This apparent contradiction could be due to stricter deadlines, greater editorial oversight, or stronger motivation to review for prestigious journals (Global State of Peer Review, 2018).

Limited access to formal peer review training further constrains system capacity and quality. In the 2018 Publons survey, 88% of respondents considered training important for ensuring high-quality peer review (Global State of Peer Review, 2018), yet peer review is not traditionally included in medical or graduate education (Kusumoto et al., 2023). Entry into peer reviewing often depends on informal pathways: 41% of survey respondents reported receiving their first review invitation only after being identified by an editor as a corresponding author (Global State of Peer Review, 2018). This mechanism systematically disadvantages ECRs, who are less likely to hold senior authorship positions and therefore remain excluded from reviewer pools.

## The impact on early career researchers

Long review timelines slow the dissemination of knowledge and delay the translation of findings into practice. In rapidly evolving fields such as global health, slow reviews can render publications outdated, undermining their potential to inform programmes and policy-making. More overlooked are the consequences of delays for individual researchers, especially at early career stages.

For ECRs, publication delays are not merely inconvenient: they carry practical, scientific, and personal costs. While slow decisions frustrate any author, for ECRs they can directly derail career progression, as timely publications are essential to secure academic positions, grants, and fellowships. In particular, early career funding schemes rely heavily on recent publications as indicators of productivity and independence. When manuscripts remain under review for extended periods, ECRs may be unable to demonstrate sufficient output, resulting in lost opportunities for essential financial support, missed or weaker grant applications, postponed graduation, or interruptions to contract continuity on research programs (Ellaway, 2024).

Prolonged review timelines also increase the risk of being scooped, whereby similar findings are published first by competitors, eroding the perceived novelty of the work and potentially leading to rejection on grounds of originality. Graduate students and postdoctoral fellows are particularly vulnerable to those consequences within an academic landscape already characterized by reduced institutional funding and growing competition (Beecher and Wang, 2025; 2019).

The mental health impact of delayed publication is equally significant. Several recent studies have identified isolation, heavy workload, and highly competitive academic environments as major drivers of psychological distress among PhD candidates and ECRs (Mahsood et al., 2025; Hall, 2024). When career advancement depends heavily on publication output, citation metrics, and journal impact factors, prolonged review timelines amplify stress, uncertainty, and feelings of professional precarity (Ellaway, 2024).

Furthermore, delays have systemic implications for equity that disproportionally affect ECRs in low- and middle-income countries. Papers from Asia, Africa, and South America are reported to spend longer under review than comparable submissions from the Global North (Liu et al., 2023). For ECRs in low- and middle-income countries (LMICs), who often face limited funding, weaker institutional support, and fewer opportunities for international collaboration, these delays directly undermine competitiveness for grants, fellowships, and mobility schemes. Importantly, large-scale peer review data indicate that these inequities are driven by structural features of the editorial system rather than lower engagement: over 96% of editors are based in established regions and are more likely to invite reviewers from their own geographic area (Global State of Peer Review, 2018). Consequently, despite reviewers from emerging regions showing higher acceptance and completion rates, they conducted only 18.9% of ScholarOne reviews between 2013 and 2017, far below their contribution to global publication output (Global State of Peer Review, 2018). This low participation in reviewing activity further restricts professional networking and visibility, marginalizing ECRs from LMICs in research participation and topic representation.

## Possible solutions

The challenges outlined above have prompted a range of different reforms, with varying implications for ECRs.

### Preprints

During the Covid-19 pandemic, many researchers turned to preprints to enable rapid dissemination when journals could not keep pace with urgent public health needs (Fraser et al., 2021). Since then, preprint use has expanded, with over 56 active servers across multiple disciplines including medical research (Krumholz et al., 2020). Preprints could be used for grant application or to support academic promotion or tenure, offering particular benefits for ECRs. However, concerns regarding misinformation and the spreading of incorrect conclusions underscore the importance of peer review for ensuring scientific reliability (Global State of Peer Review, 2018).

### Reducing review timelines

Several interventions aim to reduce review timelines directly without compromising quality. Journals can promote rapid desk-rejection of unsuitable manuscripts, set clear deadlines for reviewers with automatic reassignment if reports are not received in time, and limit revisions to one or two rounds. Evidence suggests that shorter deadlines reduce review time without substantially affecting completion rates (Global State of Peer Review, 2018). Additionally, transparent reporting of median review times would increase accountability and help authors make informed submission choices, a benefit that is particularly valuable for ECRs navigating career deadlines. Artificial intelligence tools can further shorten timelines by automating routine tasks. Validated tools already assist with plagiarism and compliance checks (Drozdz and Ladomery, 2024). Careful extension to other tasks, such as verifying references or suggesting well-matched reviewers, can speed time to first decision, allowing reviewers to focus on substantive evaluation that requires critical thinking (Checco et al., 2021).

### Incentivising participation

Expanding the peer reviewer pool requires incentives that meaningfully increase participation. Financial incentives have been explored, with some pilot programmes showing improved reviewer acceptance rates (Adam, 2025). However, these schemes are expensive for journals and may increase publication fees, posing an additional barrier for ECRs with limited funding. Moreover, they have been shown to only marginally reduce turnaround times (Cotton et al., 2025).

Non-monetary incentives are more sustainable and widely valued by reviewers. These include free access to the journal, invitation to write editorials or public acknowledgment on social media (Kusumoto et al., 2023). Open review models, ranging from publishing reviewers' names to making full reports public, represent another strategy to improve accountability and recognition. Such transparency may elevate scholarly value of reviewing and raise review quality, but concerns persist that public reports may reduce objectivity and critical appraisal (Kusumoto et al., 2023), and named critique can feel risky for junior referees assessing senior colleagues (Aczel et al., 2025). Importantly, evidence that transparency alone reduces delays remains limited.

A particularly promising and widely supported approach lies in formal recognition of peer review as scholarly output (Adam, 2025; Kadaifci et al., 2025; Kusumoto et al., 2023). Platforms such as Web of Science Reviewer Recognition (previously Publons) or ORCID credit systems already allow reviewers to document their activity. The problem is that universities and funders seldom include these contributions in evaluation criteria for hiring or promotion. Institutions could incorporate verified, editor-rated excellent reviews as a metric of service rather than sheer volume, thereby rewarding and encouraging not only engagement but also quality (Drozdz and Ladomery, 2024). Recognizing reviewing alongside publications, teaching, and grant participation would convert invisible labor into meaningful academic credit, with particular benefit for ECRs.

### Training and co-review

Finally, expanding access to structured peer review training could enable researchers to enter the reviewer pool earlier and more equitably. Several organizations, such as BMJ or Elsevier's Research Academy, have developed formal peer review training programs (BMJ, n.d.; Elsevier, n.d.), and several *Nature Portfolio* journals now encourage co-review models, where senior reviewers involve one or two ECRs as co-reviewers, with all contributors acknowledged in the final publication (2025). These models also allow ECRs to gain hands-on experience and recognition, incentivising their participation and thereby reducing reviewer shortages. Structured training is considered to improve both the quality and efficiency of peer review (Kadaifci et al., 2025). Importantly, evidence suggests that training is most effective when delivered early in a researcher's career, before reviewing practices become entrenched (Drozdz and Ladomery, 2024).

## Conclusion

The peer review crisis cannot be separated from broader structural challenges in academia. Increasingly precarious employment conditions, unequal access to research funding, and the undervaluation of academic service have progressively eroded the capacity and motivation to engage in peer review. These factors weight on ECRs, affecting their career perspectives and mental health.

Addressing these challenges requires coordinated action. Journals must commit to transparent reporting of review times and consistent editorial standards. Universities and research councils should formally recognize peer review as part of academic performance, rewarding high-quality reviewing and mentorship of junior colleagues. Funders could reinforce accountability by supporting journals that meet reasonable benchmarks for timeliness and inclusiveness.

For ECRs, such measures could mean the difference between opportunity and attrition. The current system risks discouraging talented young scientists and silencing diverse voices from resource-constrained settings. Restoring trust will require a shared commitment across the research community to ensure that peer review is timely, equitable, and fit for the next generation of scientists.

## References

[B1] (2019). The mental health of PhD researchers demands urgent attention. Nature 575, 257–258. doi: 10.1038/d41586-019-03489-1 31723298

[B2] (2025). Crediting early-career researchers in peer review. Nat. Methods. 22, 1121–1122. doi: 10.1038/s41592-025-02738-8 40500397

[B3] Aczel B. Barwich A.-S. Diekman A. B. Fishbach A. Goldstone R. L. Gomez P. . (2025). The present and future of peer review: Ideas, interventions, and evidence. Proc. Natl. Acad. Sci. 122:2401232121. doi: 10.1073/pnas.2401232121 39869808 PMC11804526

[B4] Adam D. (2025). The peer-review crisis: how to fix an overloaded system. Nature 644, 24–27. doi: 10.1038/d41586-025-02457-2 40770444

[B5] Beecher K. Wang J. (2025). Peer reviewer fatigue, or peer reviewer refusal? Account. Res. 32, 838–844. doi: 10.1080/08989621.2025.2463977 39960139

[B6] BMJ (n.d.). Reviewer Training Materials. Available online at: https://www.bmj.com/about-bmj/resources-reviewers/training-materials (Accessed January 5, 2026).

[B7] Checco A. Bracciale L. Loreti P. Pinfield S. Bianchi G. (2021). AI-assisted peer review. Human. Soci. Sci. Commun. 8:25. doi: 10.1057/s41599-020-00703-8

[B8] Chen T.-A. Ming-Hwai L. Yu-Chun C. Tzeng-Ji C. (2024). The time from submission to publication in primary health care journals: a cross-sectional study. Publications 12:12020013. doi: 10.3390/publications12020013

[B9] Cotton C. S. Alam A. Tosta S. Buchman T. G. Maslove D. M. (2025). Effect of monetary incentives on peer review acceptance and completion: a quasi-randomized interventional trial. Crit. Care Med. 53, e1181–e9. doi: 10.1097/CCM.0000000000006637 40047491

[B10] Drozdz J. A. Ladomery M. R. (2024). The peer review process: past, present, and future. Br. J. Biomed. Sci. 81:12054. doi: 10.3389/bjbs.2024.12054 38952614 PMC11215012

[B11] Ellaway R. H. (2024). Where have all the reviewers gone? Adv. Health Sci. Educ. 29, 717–720. doi: 10.1007/s10459-024-10350-2 38864958

[B12] Elsevier (n.d.) Research Academy. Available online at: https://researcheracademy.elsevier.com/navigating-peer-review/certified-peer-reviewer-course (Accessed January 5 2026).

[B13] Fraser N. Brierley L. Dey G. Polka J. K. Pálfy M. Nanni F. . (2021). The evolving role of preprints in the dissemination of COVID-19 research and their impact on the science communication landscape. PLOS Biol. 19:e3000959. doi: 10.1371/journal.pbio.3000959 33798194 PMC8046348

[B14] Global State of Peer Review (2018). Available online at: https://publons.com/static/Publons-Global-State-Of-Peer-Review-2018.pdf (Accessed January 5, 2026).

[B15] Hall S. (2024). How PhD students and other academics are fighting the mental-health crisis in science. Nature 631, 496–498. doi: 10.1038/d41586-024-02225-8 38982249

[B16] Kadaifci C. Isikli E. Topcu Y. I. (2025). Fundamental problems in the peer-review process and stakeholders' perceptions of potential suggestions for improvement. Learn. Publish. 38:1637. doi: 10.1002/leap.1637

[B17] Krumholz H. M. Bloom T. Sever R. Rawlinson C. Inglis J. R. Ross J. S. (2020). Submissions and downloads of preprints in the first year of medRxiv. JAMA. 324, 1903–1905. doi: 10.1001/jama.2020.17529 33170232 PMC7656287

[B18] Künzli N. Berger A. Czabanowska K. Lucas R. Madarasova Geckova A. Mantwill S. . (2022). “I do not have time”—is this the end of peer review in public health sciences? Public Health Rev. 43:1605407. doi: 10.3389/phrs.2022.1605407 36467128 PMC9716458

[B19] Kusumoto F. M. Bittl J. A. Creager M. A. Dauerman H. L. Lala A. McDermott M. M. . (2023). Challenges and controversies in peer review: JACC review topic of the week. J. Am. Coll. Cardiol. 82, 2054–2062. doi: 10.1016/j.jacc.2023.08.056 37968021

[B20] Liu F. Rahwan T. AlShebli B. (2023). Non-white scientists appear on fewer editorial boards, spend more time under review, and receive fewer citations. Proc. Natl. Acad. Sci. U. S. A. 120:e2215324120. doi: 10.1073/pnas.2215324120 36940343 PMC10068789

[B21] Mahsood N. Mahboob U. Asim M. Shaheen N. (2025). Assessing the well-being of PhD scholars: a scoping review. BMC Psychol. 13:362. doi: 10.1186/s40359-025-02668-2 40211401 PMC11984241

[B22] Odone A. Galea S. Stuckler D. Signorelli C. (2020). The first 10 000 COVID-19 papers in perspective: are we publishing what we should be publishing? Eur. J. Public Health. 30, 849–850. doi: 10.1093/eurpub/ckaa170 32818266 PMC7454520

[B23] Peterson C. J. Orticio C. Nugent K. (2022). The challenge of recruiting peer reviewers from one medical journal's perspective. Proc (Bayl. Univ. Med. Cent.).35, 394–396. doi: 10.1080/08998280.2022.2035189 35518802 PMC9037472

[B24] Phillips K. A. Horn D. M. (2025). Review and publication times and reporting across journals on health policy. JAMA Netw. Open. 8:e2512545. doi: 10.1001/jamanetworkopen.2025.12545 40423974 PMC12117454

